# Modulating cytokine microenvironment during T cell activation induces protective RSV-specific lung resident memory T cells in early life in mice

**DOI:** 10.1038/s44298-024-00073-x

**Published:** 2024-12-31

**Authors:** Ziyin Wang, Miko Zhong, Chubicka Thomas, Ekaterina Kinnear, Tom Rice, Beth Holder, Beate Kampmann, John S. Tregoning

**Affiliations:** 1 Department of Infectious Disease, Imperial College London, London, SW7 2AZ UK; 2 Department of Metabolism, Digestion and Reproduction, Imperial College London, London, W12 0NN UK; 3 Centre for Global Health, Charité Universitatsmedizin, Berlin, Germany; 4 Present Address: Centre for Endocrinology, Queen Mary University of London, London, UK

**Keywords:** Vaccines, Immunopathogenesis

## Abstract

Maternal immunisation against respiratory viruses provides protection in early life, but as antibodies wane, there can be a gap in coverage. This immunity gap might be filled by inducing pathogen-specific lung tissue-resident T cells (TRM). However, the neonatal mouse lung has a different inflammatory environment to the adult lung which affects T cell recruitment. We compared the factors affecting viral-specific TRM recruitment in the lungs of adult or neonatal mice. In contrast to adulthood, we demonstrated that RSV or influenza infection in neonatal mice recruited fewer TRM to the lungs. This was associated with reduced lung levels of CCL5 and CXCL10. Co-administration of CCL5 or CXCL10 at the time of primary T cell activation significantly increased RSV-specific TRM in the lung, protecting mice upon reinfection. These chemokine differences were reflected in responses to infection in human cord blood. Here we show a critical role for CCL5 and CXCL10 in the induction of lung TRM and a possible strategy for boosting responses.

## Introduction

RSV causes 200,000 deaths yearly, with a disproportionate burden in low-income countries^1^; in the last few years, there have been dramatic advances in the development and deployment of RSV vaccines^2^. For infants, RSV vaccines provide passive protection following maternal immunisation^3^; the alternative approach is passive protection through monoclonal antibody administration^4^. In the UK, a decision has been made to implement the maternal vaccine^5^, but other countries, for example, Spain, are using universally administered monoclonal antibody^6^. Both are attractive strategies and will serve to reduce infection in the first 6 months of life. But they are not the complete solution to protect children from RSV disease, the major challenge of both approaches is that because they rely on passive immunity, protection will wane. In the absence of vaccination, levels of transferred maternal antibody decline rapidly in the infant; one study modelled the approximate half-life to be 35 days projecting the duration of response was 4.7 months^7^; this is similar to other antigens^8^. So even if the vaccine elevates maternal antibody titres^9^, there is still a window of susceptibility in the infant; this is of particular concern in premature infants who may only receive a smaller amount of maternal antibody and could still be developmentally immature as the antibody wanes. Questions remain about whether delaying RSV infection to the second year of life will simply delay the peak of the disease. For monoclonal antibody therapy, there is an additional risk of viral mutation and escape^10^. Therefore, it could be beneficial to boost immune protection to the child.

One approach to extend protection against RSV would be to boost the antibody response by vaccinating at the low ebb of antibodies. However, maternal vaccination can attenuate subsequent antibody responses in the child, for example, after measles^11^ or pertussis^12^ vaccination. An alternative strategy is to induce protective T cells with a vaccine because they have been shown to correlate with protection against viral lung infection^13^. A tissue-resident subset of T cells (TRM) has recently been identified as a key component of protective cellular immunity^14^. TRM cells act as sentinels at mucosal sites responding rapidly to infection, leading to an antiviral response^15^. These cells are derived from circulating effector T cells that migrate into tissues and, under key transcription factors (Hobit/Blimp), lose receptors that enable tissue egress (CCR7, S1PR1) and gain integrins (αE/β7) that enable tissue retention^14^. TRM cells can be defined by the expression of cell surface markers, including the activation marker, CD69, and the integrin, CD103. TRM cells are found in the lungs after human RSV infection, and their numbers correlate with protection against challenge^16^. We^17^ and others^18^ have demonstrated that TRM cells are sufficient to protect against RSV infection, and it has been shown that vaccine-induced RSV-specific TRM is protective against viral infection^19^. Based on this, we believe that tissue-resident memory T cells could be a critical target for an RSV vaccine to be administered between 6 and 12 months of life.

However, in order to generate protective T cells after vaccination, we need to understand more about the induction of TRM and, specifically, the requirements to induce TRM in early life. Here we explored the immune response in very early life; the immune system of neonates is developmentally adapted and differentially regulated. We have shown that RSV infection in neonatal (7-day-old) mice induces antigen-specific T cells, but the neonatal memory T cell response is different from that in adult mice^20–22^. A similar long-term imprinting effect has been seen on human T cells after RSV infection in the first year of life^23^. A recent study has observed significantly fewer TRM in the lungs of neonatal mice following RSV infection compared to adult mice^24^. Whilst the mechanisms by which TRM develop in the adult lungs are beginning to be established^15^, why or how the primary immune response fails to generate robust TRM in children remains unclear. There is also extensive evidence for the involvement of chemokines in the lung to selectively recruit inflammatory cells, such as CCL2 and CCL5, in both mouse models^25^ and human studies^26^. Here, we show that mice primed at 7 days of age with RSV and CCL5 or CXCL10 made more TRM when rechallenged with RSV as adults. This could be a potential way to boost TRM recruitment through vaccination.

## Results

### Primary RSV infection induces a differential response in neonates resulting in a paucity of TRM being produced

**Fig. 1:**
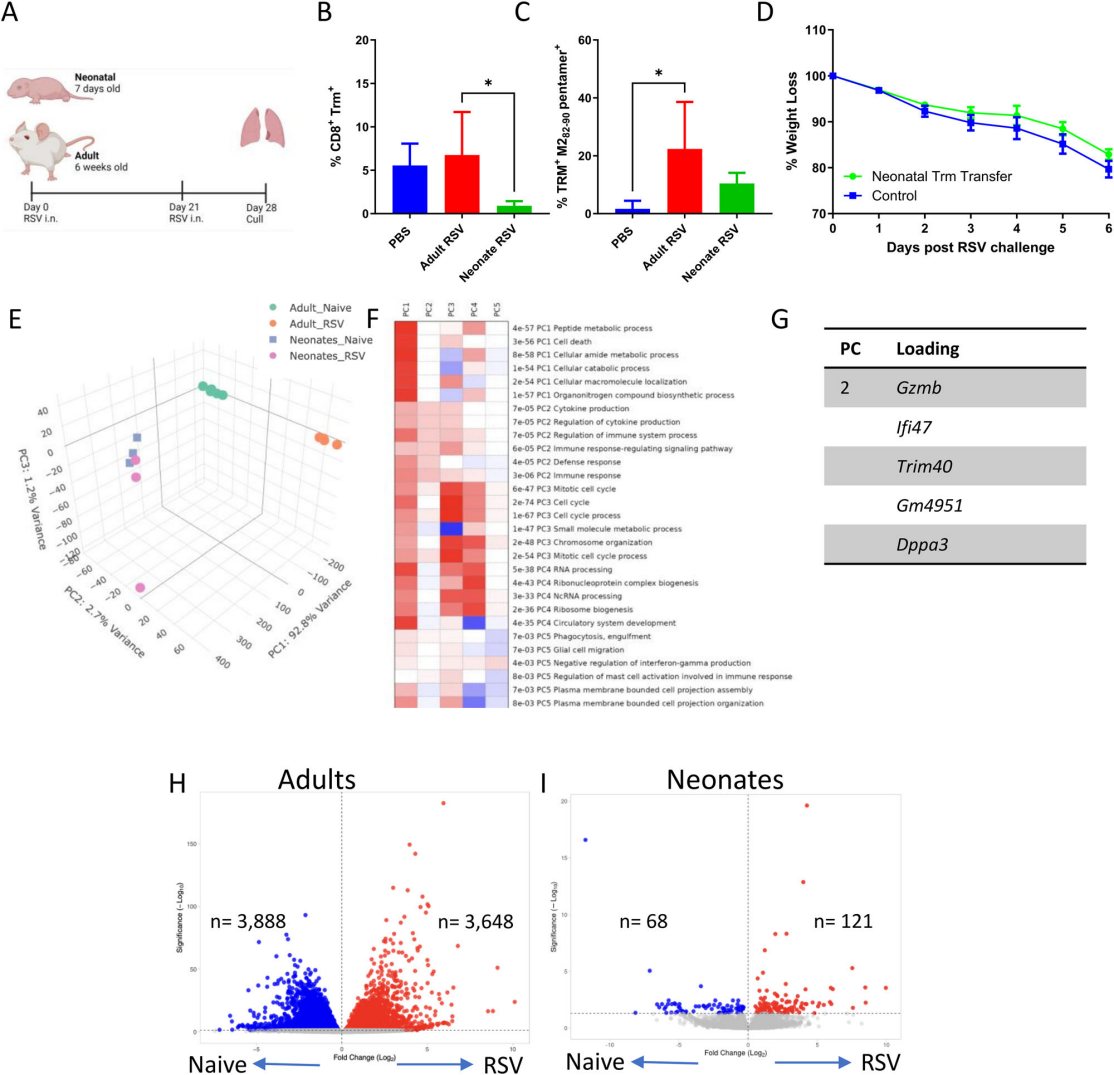
Differential lung response induced by RSV in neonates compared to adults. Female 6–7 weeks old (adult) or mixed 7 days old (neonates) BALB/c mice were infected with RSV. Infection schedule made with BioRender (**A**). Percentage all CD8 Trm (**B)** and RSV specific (**C**). Airway cells were transferred from neonatal RSV-infected mice into naïve adult mice prior to the RSV challenge of the recipient mouse, weight at d7 (**D**). In a separate study, neonatal and adult mice were infected with RSV, on day 7, lungs were collected from the infected animals and RNAseq was carried out from RNA extracted. PCA of genes in different groups (**E)**, pathways related to each PC (**F**) and loading genes driving PC2 and PC3 are shown in (**G**). Volcano plots of DEG from Adult (**H**) and Neonatal (**I**) mice. Bars in **B** and **C** represent mean ± SEM of *n* = 5 mice. *p < 0.05.

To confirm previous studies^24^, we compared the effect of age on levels of lung CD8 TRM after RSV infection in a mouse model. Seven-day-old (neonates) or 6-week-old BALB/c (adult) were infected with RSV A2 virus and sacrificed 21 days after infection (Fig. 1A). There was a significantly greater proportion of CD69^+^/CD103^+^ CD8^+^ T cells in the lungs of adult mice (Fig. 1B), and significantly more of these were specific for the RSV M2_82–90_ pentamer (Fig. 1C). Previously we have observed that transferring airway cells from RSV infected adult mice to naïve mice was protective against subsequent RSV infection^17^. When we transferred cells from the airways of mice infected with RSV as neonates, there was no protective effect (Fig. 1D), indicating there was no localised T cell protection after RSV infection in neonatal mice.

Having observed reduced TRM following neonatal RSV infection, we used RNASeq to identify whether there were transcriptomic differences in the lung that might explain the different recruitment of TRM to the lungs. We first evaluated global transcriptomic responses in lung RNA following RSV infection in neonatal and adult mice. Principal component analysis (PCA) demonstrated clear separation between the adult and neonatal groups; this was driven predominantly by PC1 (which accounted for 92.8% of the variance). There was also a distinct separation in the transcriptomic profile induced upon RSV infection in adult mice (Fig. 1E). This separation was predominately driven by PC2, marked by cytokine production (*p* < 7^−05^), immune response-regulating signalling pathway (*p* < 5^−^^05^), and defence response (*p* < 4^−05^) (Fig. 1F). There was minimal separation between the infected and uninfected neonatal groups by PCA. Loading genes contributing to PC2 included granzyme B (*Gzmb*), interferon-gamma inducible protein 47 (*Ifi47*), ubiquitin ligase (*Trim40*), immunity-related GTPase (*Gm4951*), and developmental pluripotency-associated protein 3 (*Dppa3*) (Fig. 1G). There were clear differences in the numbers of differentially expressed genes (DEGs) between adult (Fig. 1H) and neonatal (Fig. 1I) mice; adult mice had a total of 7536 DEGs, whereas there was 189 DEGs identified in neonates blood transcriptomics.

### Poor accumulation of TRM is associated with reduced levels of MCP-1, CCL5, and GM-CSF

**Fig. 2:**
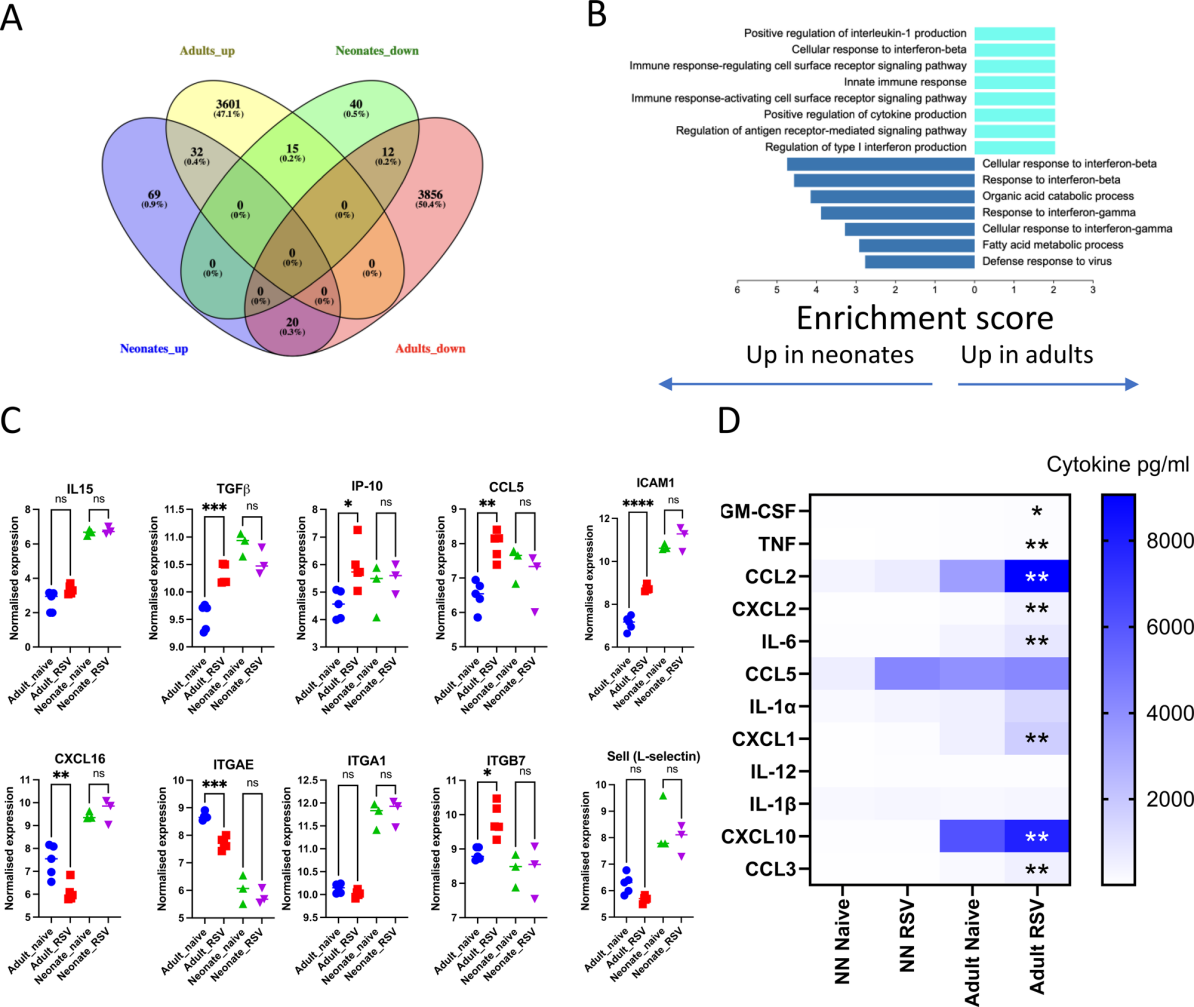
Neonatal RSV infection induces a less pronounced cytokine response than adults. Venn diagram showing the overlaps between neonates and adult sequencing data (**A**). KEGG Pathways upregulated in neonates and adults (**B**). Relative expression level of selected genes (**C**). Cytokines measured in the lung in adult or neonatal mice 24 h after RSV infection; *p < 0.05, **p < 0.01 compared between Ad RSV and NN RSV (**D**).

Having observed significantly different transcriptomic profiles in response to RSV infection in the lungs of different aged mice, we explored the types of genes associated with the differences. When the two groups were compared (Fig. 2A), there were 32 genes both upregulated in adults and neonates, these related to complement pathways (*C1s1, C1ra*), apoptosis (*Casp12*), platelet homoeostasis (*Nos2, Nos3, Nos1, Gucy1b1, Gucy1a1, Gucy1a2, Atp2b4, Clu*) and interferon signalling pathway (*Usp18, Oas2, Ifit1, Ifit3*). When the DEGs were grouped by GO terms, there was a clear increase in cytokine-associated pathways in adult mice (Fig. 2B). Upregulated pathways in adult lung RNA following RSV infection included positive regulation of interleukin-1 production, positive regulation of cytokine production and regulation of antigen receptor-mediated signalling pathway. In comparison, the top enriched pathways in neonates were interferon-beta and gamma-focused.

We next explored the cytokine gene transcripts from the lung transcriptomics, focusing on genes associated with TRM recruitment (the cytokine genes *Il15* and *Tgfb*, and the chemokine genes *Cxcl10* (IP-10), *Ccl5* (RANTES) and *Cxcl16*) and retention (integrins *Itgae*, *Itga1* and *Itgb7*). Of these, *Tgfb*, *Cxcl10*, *Ccl5* and *Itgb7* were significantly increased in adult lungs after RSV infection compared to age-matched control. Both *Cxcl16* and *Itgae* were decreased significantly in adults. *Il15* and *Itga1* did not show any change in either adults or neonates. No significant increase in any of the TRM-related genes was observed in neonates (Fig. 2C). We then looked at protein levels of cytokines in the lung at an acute time point after infection. There was significantly more GM-CSF, CCL2, CXCL2, IL-6, CXCL1, CXCL10 and CCL3 in the lungs of adult infected mice compared to infected neonatal mice (Fig. 2D); CCL5 protein levels were elevated after infection in both ages, but there was no difference between ages of mice.

### Cytokine responses to RSV in PBMC from cord blood were similar to adult blood

**Fig. 3:**
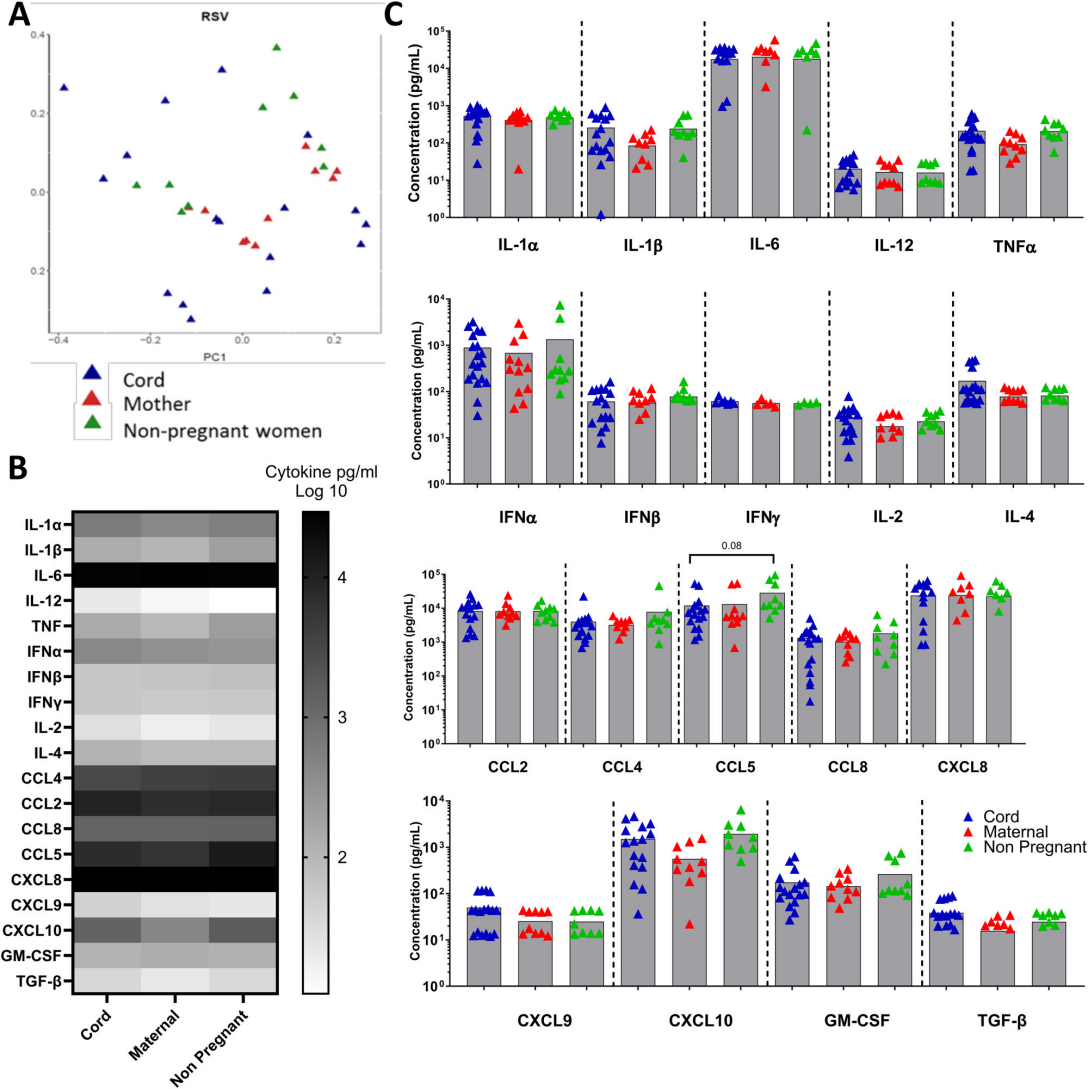
Cytokines response to RSV stimulation in cells from mums, babies and non-pregnant women. PBMC isolated from cord blood, maternal blood post-partum and non-pregnant mothers were stimulated with RSV for 24 h. Cytokines were measured in the supernatant by Luminex. PCA analysis of all data points (**A**). Heat map of cytokine responses (**B**). Individual cytokine responses (**C**). Number of donors, cord blood *n* = 21, maternal *n* = 13 and non-pregnant *n* = 9.

Having seen age-related differences in the murine response to RSV we wanted to compare the human response. As a model of neonatal human immune responses, we used cord blood; we compared the responses to the mothers post-partum and to non-pregnant women to account for the immuno-modulatory effect of pregnancy. PBMC isolated from cord blood (*n* = 18), blood from mothers post-partum (*n* = 13) or non-pregnant women (*n* = 9) were incubated with RSV for 24 h. The cord blood and maternal blood came from the same mother–baby pair. Supernatants from these stimulations were collected and analysed by Luminex or pan-IFNα ELISA to investigate the immune response profile. To compare overall patterns of response, principal component analysis (PCA) was used to compress and transform the multivariate Luminex data. There were no significant differences in the global cytokine profiles between cord blood, mothers, and non-pregnant women (Fig. 3A). All three populations had moderate to high levels of inflammatory cytokines expression after RSV infection in vitro (Fig. 3B). There was no significant difference between the three groups of donors in individual inflammatory cytokines (Fig. 3C); however, there was a trend towards CCL5 being lower in the cord blood.

### Increasing levels of CCL5 and CXCL10 after neonatal RSV infection boosts protective lung TRM on re-challenge

**Fig. 4:**
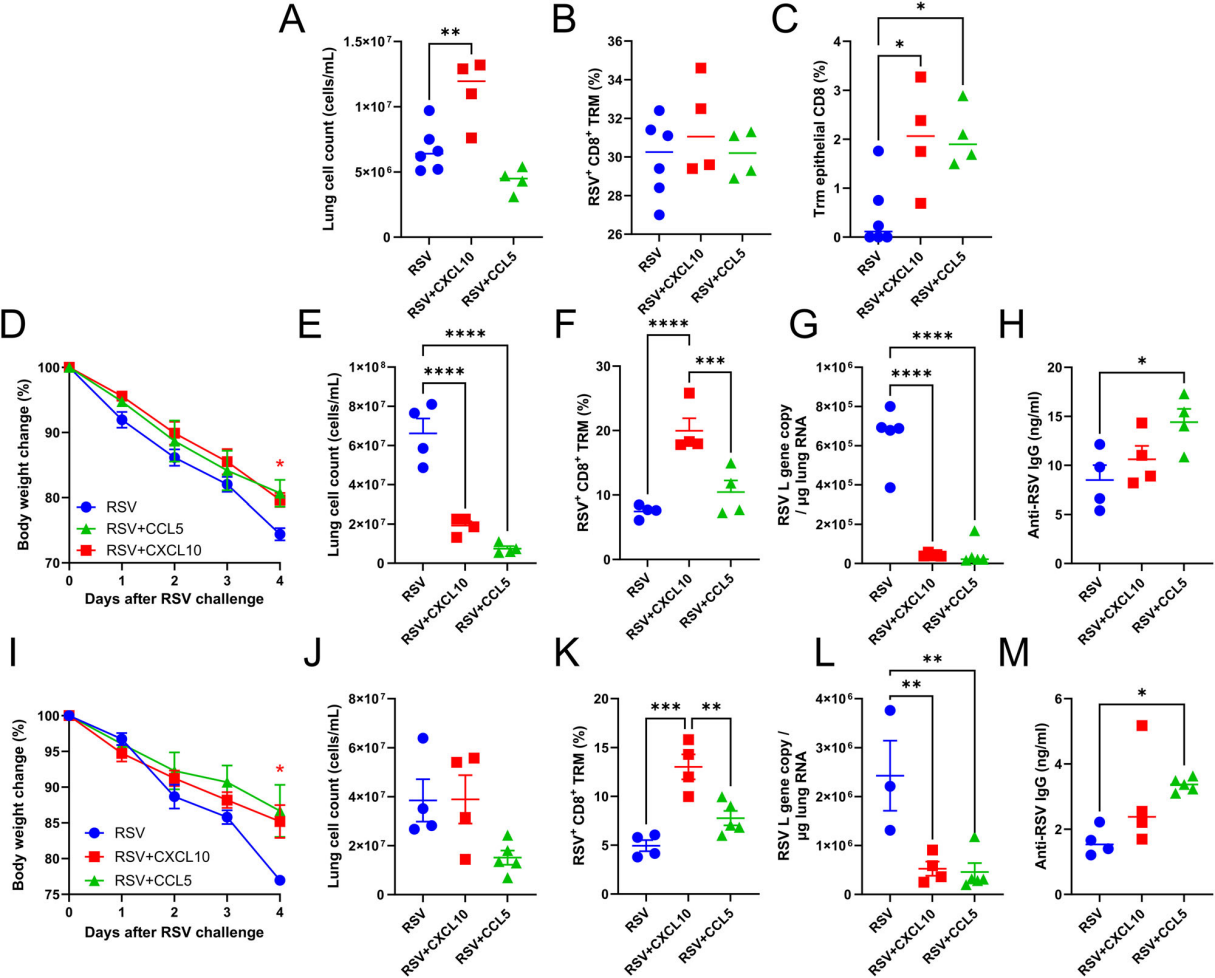
Boosting primary RSV infection with chemokines enhanced tissue resident TRM production and confer protection. Seven-day-old BALB/c mice were infected intranasally with RSV, and 20 µg of CCL5 or CXCL10 in 20 μl were given on days 7, 8 and 9 after infection. Mice were culled on day 21, and flow cytometry was performed on the lungs that were harvested from the animals. Lung cell count (**A**), CD8 (**B**), Trm epithelial CD8 T cells (**C**). In a separate study, neonatal mice were infected with RSV and subsequently received chemokines intranasally on d7, 8 and 9 of infection before re-challenge on day 21. Mice were followed for 4 days after infection, and weight loss (**D**), lung cell count (**E**), CD8 TRM% (**F**), Viral Load (**G**) and antibody (**H**) were recorded. The same set-up was repeated except CCL5 or CXCL10 was given only on day 7 post primary infection. Mice were followed for 4 days after infection, and weight loss (**I**), lung cell count (**J**), CD8 TRM% (**K**), Viral Load (**L**) and antibody (**M**) were recorded. *p < 0.05, **p < 0.01, ***p < 0.001; statistical analysis by one-way ANOVA except for panels **D** and **I** where 2 way ANOVA. *N* = 5 mice per study.

Whilst we saw a difference in expression levels of a range of cytokines, previous studies have shown associations between CCL5 and CXCL10 and the recruitment of TRM^14^; therefore, we investigated the effect of administering recombinant CCL5 and CXCL10 into neonatal lungs of mice at the peak of T cell response, day 7–9 after RSV infection (Fig. S2A). We focussed on this time point because that is the peak of T cell recruitment after RSV infection^21^. Seven-day old BALB/c mice were infected intranasally with RSV; 20 µg of CCL5 or CXCL10 in 20 μl were given on days 7, 8 and 9 after infection. Mice were culled on day 21, and flow cytometry was performed on lungs harvested from the animals. Mice treated with CXCL10 had significantly more cells recruited into the lungs after infection (Fig. 4A). Although there was no difference in the number of CD8 T cells (Fig. 4B), central memory T cells or non-epithelial TRM cells (RSV^+^CD8^+^CD69^+^CD103^−^), there was a significant increase in the proportion of tissue-resident TRM cells (RSV^+^CD8^+^CD69^+^CD103^+^) after CXCL10 and CCL5 treatment (Fig. 4C).

We and others have previously observed that RSV infection in neonatal mice primes for more severe disease on re-infection, and this is driven by the recruitment of CD8 T cells during secondary infection^21^. We speculate that this is, in part, caused by the absence of TRM following neonatal RSV infection. Having seen increased CD8 TRM in the lung after the addition of chemokines, we investigated whether the administration of chemokines had an impact on disease following re-challenge with RSV (Fig. S2B). As previously seen, the control RSV group lost 15–20% of their original body weight on day 4 after re-challenge (Fig. 4D) and also recruited significantly more cells to the lung compared to CXCL10 or CCL5 treated mice (Fig. 4E). Although there was no difference in the percentage of CD8^+^ T cells in the lung, mice treated with CXCL10 had significantly increased tissue-resident TRM cells (Fig. 4F). The reduced disease was reflected by a significantly reduced viral load (Fig. 4G). Interestingly the addition of CCL5 significantly increased the amount of RSV-specific antibody in the sera (Fig. 4H).

Having seen that the addition of chemokines over 3 days during the peak of T cell recruitment altered the response to RSV infection and protection against secondary re-infection, we explored if a single dose of CCL5 or CXCL10 on day 7 after infection would be sufficient to enhance TRM recruitment (Fig. S2C). As with dosing over 3 days, CXCL10 addition at day 7 after neonatal RSV infection significantly reduced weight loss on RSV rechallenge of neonatally primed mice (Fig. 4I). There was no difference in lung cell recruitment between the three groups (Fig. 4J). CXCL10 treatment, but not CCL5, enhanced tissue resident TRM count in the lung (Fig. 4K). Both treatment groups had significantly less RSV L gene copy in the lung (Fig. 4L). As with 3 doses, there was more RSV-specific IgG in the serum collected from CCL5-treated mice (Fig. 4M), suggesting a different mechanism of protection in those mice against weight loss.

### Cytokine and TRM response to neonatal influenza virus infection is also blunted

**Fig. 5:**
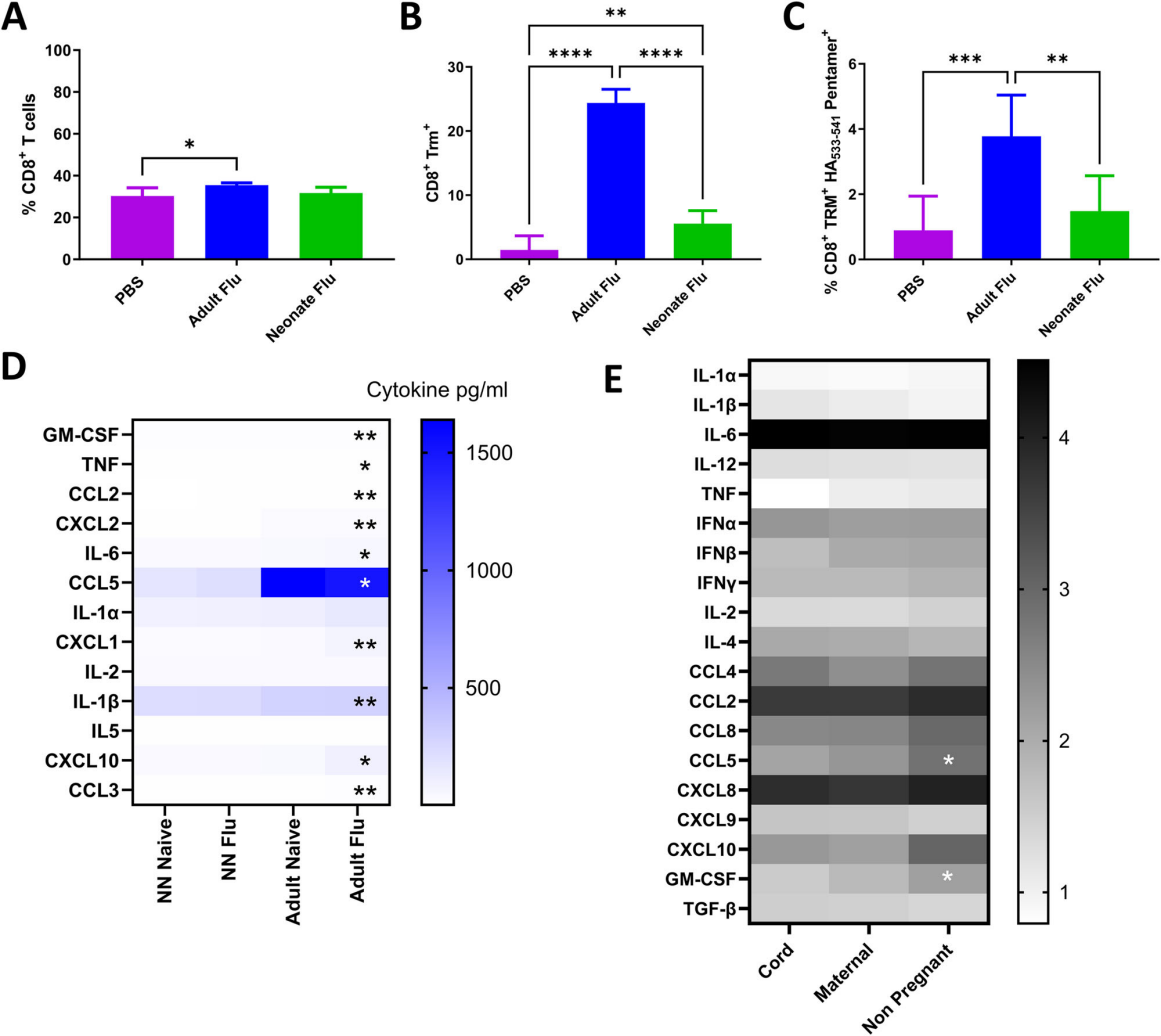
Neonatal response to influenza virus is also blunted. Female 6–7 weeks old (adult) or mixed 7 days old (neonates) BALB/c mice were infected with RSV. Percentage all CD8 (**A**), Trm (**B**) and RSV specific (**C**). Cytokines were measured in the lungs in adult or neonatal mice 24 h after influenza virus infection (**D**). PBMC isolated from cord blood, maternal blood post-partum and non-pregnant mothers were stimulated with RSV for 24 h. Cytokines were measured in the supernatant by Luminex (**E**).

We wanted to see if the failure to generate TRM following neonatal infection was a conserved response to viral infection in early life. A similar phenotype of reduced lung TRM induction has recently been observed following neonatal influenza infection^27^. As with RSV, we infected 7-day and 7-week-old mice with the H1N1 influenza virus intranasally and measured the immune response in the lung 21 days later. The proportion of CD8 T cells in the lung was significantly greater in adult mice than in naïve mice (Fig. 5A). There was a significant increase in total CD8 TRM as a proportion of cells recovered from the lung (Fig. 5B), and they were influenza-specific (HA_533–541_ Fig. 5C). We also evaluated the cytokine response to infection in lung 24 h after infection. The levels of GM-CSF, TNF, CCL2, CXCL2, IL-6, CCL5, CXCL1, IL-1β, CXCL10 and CCL3 in the lungs were significantly higher in infected adult lungs than infected neonatal mice (Fig. 5D). We then compared the response to influenza in human PBMC, using the same system described above. There was a significantly higher level of CCL5 and GM-CSF after live influenza virus stimulation of PBMC from non-pregnant women compared to cord blood (Fig. 5E).

One of the confounding factors for studying the cytokine response to viral infection might be pre-existing antibodies. The majority of adults have encountered RSV and influenza infection at some point. This could also affect neonates, because neonatal antibody is mostly of maternal origin due to the transplacental transfer. Therefore, we tested whether pre-existing antibodies have a significant impact on cytokine responses to the influenza virus in this experimental setup. H1N1-specific IgG were measured in plasma samples by antigen-specific ELISA. No correlation was found between IFNβ or IL-2 responses and H1N1 specific IgG in either cord or mothers (IFNβ: *r*^2^_cord_ = 0.221, *r*^2^_mother_ = 0.258; IL-2: *r*^2^_cord_ < 0.01, *r*^2^_mother_ = 0.202). There was no correlation between either CCL5 or CXCL10 response to the influenza virus, and pre-existing H1N1 specific IgG in both cord and maternal blood samples (CCL5: *r*^2^_cord_ = 0.132, *r*^2^_mother_ = 0.068; CXCL10: *r*^2^_cord_ = 0.117, *r*^2^_mother_ = 0.296). The GM-CSF response to the influenza virus was not correlated with pre-existing H1N1-specific IgG titre in neonates and their mothers (*r*^2^_cord_ = 0.097, *r*^2^_mother_ = 0.011).

## Discussion

In the current study, we explored the role of chemokines after neonatal respiratory viral infection in mice, in order to understand the reduced generation of TRM. As with previous studies, reduced levels of TRM after neonatal RSV^24^ or influenza virus^27^ infection were observed. RNA-Seq analysis of lungs from RSV-infected mice showed a significantly different gene expression profile, with a greater magnitude response in the adult mice, as observed previously^28^. It was notable that the neonatal mice had a much lower number of DEG, further exploration of why this occurs is needed. The DEG in the lungs after adult infection were clustered in cytokine pathways. This was reflected by increased protein levels of some of the cytokines in the lungs; though CCL5 had increased transcript, there was a difference in protein. Whilst we saw no difference after stimulation of cord blood and adult derived PBMC with RSV, there was significantly less CCL5 and GM-CSF produced following influenza virus stimulation of cord blood cells. When the TRM-associated chemokine CXCL10 was delivered intranasally at the peak of T cell recruitment to the lung during RSV infection, there was a change in the profile of memory CD8 cells that was associated with reduced weight loss following RSV re-challenge of neonatally primed mice. This suggests that there is a key deficit in one or more chemokines required to recruit TRM to the lungs of neonatal mice after viral infection.

Why this deficit in chemokine production and cell recruitment occurs needs more investigation. Respiratory (CD103^+^) dendritic cells but not CD11b^+^ DC have been shown to be important in TRM programming, and a recent study described a defect in this key DC subset in neonatal lungs^29^. Boosting the CD103 DC population with Flt3 ligand increased CD69 expression on neonatal CD8 cells after RSV infection^28^; likewise, co-administering RSV with CpG increased the numbers of CD8 TRM in the lungs^24^. This mirrors the necessity of type I IFN during adult infection for the induction of adult TRM^30^. Another possibility is that the neonatal lungs lack adhesion molecules for the retention of Trm, we saw differential expression of integrins between adult and neonatal lungs, lower levels of *Icam1* have been observed in a previous study^31^. Another possible mechanism that may affect Trm survival is altered cell metabolism. Resident memory cells utilise different energy pathways compared to central memory cells, for example Trm have been shown to require free fatty acids to survive^32^; whether neonatal lungs provide this environment needs further investigation. One way to test whether the effect is at recruitment or retention would be to transfer adult Trm into neonatal mice prior to infection, we have seen this to be protective when performed between adult mice^17^. This effect is likely also to be affected by host genotype, we have previously observed that mouse haplotype plays a key role in the delayed effects of neonatal RSV infection^22^, why the CD8 T cell–DC interaction in some inbred strains leads to a more potent response needs further evaluation. The pauci-responsiveness of neonatal DC viral infection may be a protective adaptation to the acute onset of novel antigens in the transition from the womb to breathing in air. Previous studies have shown that by weaning age, the effect is less marked, so it may also be influenced by changes in diet^33^. Recent studies have observed dynamic changes in the proteome in the first weeks of life, reflecting adaptation to the post-partum environment^34^. Notably, levels of CXCL10 increase in the plasma of an infant cohort over the first 7 days of life^35^.

When we supplemented the response with CXCL10, there was increased TRM recruitment to the lungs and protection against viral re-infection. This indicates that it is possible to recruit TRM to the lungs in early life and retain them there. CCL5 and CXCL10 have previously been identified to be important in the recruitment of Trm to the lungs in an influenza model^14^. Whilst we have previously observed that CD8 cells are recruited to the neonatal lung after RSV infection, this was significantly lower than in adult mice^21^. How this could be applied to future vaccination strategies is an important question. Mucosal T cells have been proposed to potentially reduce infection and onward transmission of viral infections or at least as part of a layered adaptive immune response^36^. More generally the question of how to recruit mucosal T cells following vaccination needs addressing. Given these findings, intranasal live-attenuated viral vaccines could have great potential as a booster strategy for RSV vaccines. In the development of RSV vaccines, there have been challenges with getting the balance right: generating a virus that is sufficiently attenuated not to cause disease but not so attenuated that it is not able to replicate in the human airways. One approach to generate live attenuated RSV is to recode the genome use codon pair deoptimisation^37^, this has been applied to RSV^38^. But the main approach has been gene deletion and the adaptation of temperature-sensitive mutants a number of these are in clinical trials in young children 6–24 months^39,40^. Ensuring that these vaccines trigger the right type of response to recruit TRM will be important in maximising their protective efficacy.

## Methods

### Mice

Adult (6–8 week-old) or Neonatal (7 day-old) BALB/c mice were obtained from Charles River Ltd. (St Mary’s, UK) and maintained according to institutional and Home Office guidelines. All experiments were performed in the SPF room in the animal facility on a 12-h light/dark cycle at 20–24 °C with 55% ± 10% humidity at Imperial College London, St Mary’s Hospital Campus. All work was approved by the Animal Welfare and Ethical Review Board at Imperial College London, and studies were in accordance with the Animal Research: Reporting of In vivo Experiments (ARRIVE) guidelines.

Mice were housed in groups of five animals per cage. Sample sizes were calculated using the G*Power raw software package, based on previously generated data for weight loss in the same model; weight loss was the primary outcome measure. No criteria were set for including and excluding animals. For RSV infection studies, mice were anaesthetised using 2.5–3% of isoflurane and intranasally (i.n.) infected with 10^6^ PFU in 100 μl (adults) or 2 × 10^5^ PFU in 20 μl (neonates) RSV subgroup A2 as indicated in the figure legend. For chemokine boosting, mice received 20 μg in 20 μl of CCL5 or CXCL10 i.n. under anaesthesia on d7, 8 and 9 after initial RSV infection, as indicated in the figure legend. In re-challenge studies, mice were re-infected with 5 × 10^6^ RSV 21 days after the initial infection.

For influenza infection, mice were infected under anaesthesia with 5 × 10^4^ PFU in 100 μl (adults) or 400 PFU in 20 μl (Neonates) H1N1/Eng/195.

### Lungs and airway cell isolation

On day 4 of the re-challenge, mice were culled 10 min after intravenous (i.v.) injection with 2 μg (in 200 μl) of PE-labelled anti-CD45 antibody (Cat!). Mice were culled using 100 μl intraperitoneal pentobarbitone (20 mg dose, Pentoject, Animalcare Ltd. UK). Lung tissue and BAL were collected as previously described^41^. Lungs were homogenised by passage through 100 μm cell strainers, then centrifuged at 1500 rpm for 5 min. Supernatants were removed, and the cell pellet was treated with red blood cell lysis buffer (ACK; 0.15 M ammonium chloride, 1 M potassium hydrogen carbonate, and 0.01 mM EDTA, pH 7.2) before centrifugation at 1500 rpm for 5 min. The remaining cells were resuspended in RPMI 1640 medium with 10% foetal calf serum, and viable cell numbers were determined by trypan blue exclusion.

### Flow cytometry

Live lung cells and cells from BAL were plated out onto a U-shaped 96-well plate and then spun down at 2000 rpm for 2 min at 4 °C. In total, 100 µl of Live/Dead violet dye (ArCTM, Catalogue: A10346) was added for 20 min at 4 °C in the dark, the plate was then centrifuged at 2000 rpm for 2 min, and the supernatant was taken off. The cell pellet was resuspended in Fc block (Clone: 2.4G2) in PBS-1% BSA and stained with the following surface antibodies: FITC anti-mouse CD3 (Clone: 12A2, Cat: 100204, BioLegend), APC-H7 anti-CD8 (Clone: 53-6.7, Cat: 560247 BD Biosciences), BV605 anti-CD103 (Clone: 2E7, Cat: 121433 BioLegend), APC anti-mouse CD69 (Clone: H1.2F3, Cat: 104513 BioLegend), PerCP-Cy5.5 anti-mouse CD4 (Clone: RM4-5, Cat: 100540 BioLegend), BV711 anti-mouse CD44 (Clone: IM7, Cat: 103057 BioLegend), PE-Cy7 anti-mouse CD62L (Clone: MEL-14, Cat: 104418 BioLegend) for one hour in the dark. Excess antibodies were washed off with 1% BSA in PBS three times before being filtered through the FAC tubes on an LSR Fortessa Flow cytometer (BD) and FlowJo. Fluorescent minus one (FMO) controls were used for surface stains. Analysis was performed using FlowJo and gated, as shown in Fig. S1.

### Airway cell transfer

Cells were collected from BAL, washed and resuspended in sterile PBS. Mice were anaesthetized, and 10^6^ cells in 100 μl were delivered intranasally with a Gilson pipette.

### qPCR

RNA was extracted from the left lung lobe by first homogenising the tissue using a TissueLyzer (Qiagen, Manchester, UK) at 50 oscillations for 4 min followed by a TRIzol (QIAzol, 79306; Qiagen) and chloroform extraction. RNA concentrations were determined using a Nanodrop before converting into cDNA using a GoScript reverse transcription system (Product code A5001; Promega, UK) and 2 µg for all samples. qPCR for the RSV L gene was performed on a Stratagene Mx 3005p (Agilent Technologies, Santa Clara, CA, USA) using the primers 5′-GAACTCAGTGTAGGTAGAATGTTTGCA-3′ and 5′-TTCAGCTATCATTTTCTCTGCCAA-3′ and probe 5′-6-carboxyfluorescein (FAM)-TTTGAACCTGTCTGAACAT-6-carboxytetramethylrhodamine (TAMRA)-3′. RNA copy number per mg of lung RNA was determined using an RSV L gene standard. Gene expression levels of the RSV L gene were normalised to the GAPDH copy number.

### RNA extraction, processing and normalisation

Lung RNA was extracted using QIAzol (Qiagen) and chloroform extraction. RNA QC and library preparation were performed by Novogene using the Illumina HiSeq at a target depth of 50 million 100–150 bp pair-end reads per sample. The quality of Raw RNAseq reads generated was assessed using FastQC (v0.11.9) to ensure good quality scores, GC content, and no adaptor reads, then appropriate adjustment was made using the programme Trimmomatic (v1.0.40). Raw reads were then mapped to the Mouse Reference Genome (Gmc38) using STAR (Spliced Transcripts Alignment to a Reference, v6.2.0) and count data for each gene performed using Salmon (v1.2.0). Principal component analysis (PCA) and heatmap visualisation on normalised sequence data were analysed by the variance stabilising transformation method. R function prcomp() was used for PCA in the package devtools (v2.4.2) and heatmap visualisation was performed the heatmap.2 function in gplot package (v3.1.1).

### Differential expression analysis

Differential gene expression analysis was performed using DeSeq2^42^ differential analysis to obtain a list of genes, *P* values, adjusted *P* values and log2 fold changes with positive log fold change values indicating increased gene expression and negative values decreasing gene expression. False discovery rate (FDR) was calculated by applying the weighted Benjamin–Hochberg method for multiple hypothesis testing. A gene was considered differentially expressed if the absolute fold change was above 0.5 with an adjusted *P*-value < 0.05.

### Gene ontology and KEGG network analysis

ClusterProfiler^43^ was used to assess the enrichment of Gene Ontology (GO) pathways in each gene list. Network analysis of the GO terms was analysed using the emapplot function in the clusterProfiler package. The KEGG pathway was analysed using the gseKEGG function. Gene lists analysed include genes that were identified as significantly differentially variable and genes that were in a specific GO term. Gene set enrichment analysis (GSEA) was performed where the enrichment score was calculated as the −log10 (*P*-value)^44^. The network analysis of KEGG pathways (network visualisation and clustering) was performed with NetworkAnalyst^45^. R code is available upon request.

### Multiplex cytokine assay (mouse)

Lung cytokine levels were measured using commercial multi-spot U PLEX kits from Meso Scale Discovery (MSD) and performed according to the manufacturer’s instructions. Data was analysed, and lower limits of quantification (LLOQ) were determined using MSD DISCOVERY WORKBENCH software.

### Human Cord blood

#### Recruitment

This study was a nested study within a larger study investigating maternal pertussis vaccination. It was an opportunistic study using samples from the same individuals and not specifically powered to look at the questions. Healthy pregnant women were recruited antenatally. Exclusion criteria included: twin pregnancy, maternal infection, chronic maternal pathology, pregnancy pathology, and babies with chromosomal or structural abnormalities. The study was approved by the National Research Ethics Service (NRES), NHS, UK (REC 13/LO/1712) and stored under the Imperial College Healthcare Tissue Bank (ICHTB). Written informed consent was obtained.

#### Blood sample collection

Cord blood and maternal blood were collected in sodium heparin-anti-coagulated Vacutainer tubes (BD Biosciences). Cord blood was obtained from the cords of healthy neonates at the Maternity Unit of St Mary’s Hospital, London. Through collaboration with Dr Beth Holder and Professor Beate Kampmann (Department of Paediatrics, Imperial College London), all participants had completed written informed consent, and the study was approved by the ethics committee of the Faculty of Medicine, Imperial College London. Blood samples from non-pregnant women were also collected as a comparator, these blood samples were obtained from volunteers in the Department of Infectious Disease. Maternal blood samples were collected from mothers at or within 2 days of delivery. Umbilical cord blood samples were collected at delivery.

#### Peripheral blood mononuclear cell (PBMC) isolation

Blood samples were processed no more than 8 h after birth. Blood samples were diluted with PBS in a 1:2 ratio. This mixture was then layered with an equal volume of Histopaque (density: 1.077 g/mL, Sigma-Aldrich) and centrifuged at 1000 RPM for 20 min with the brake off. The cloudy interphase layer, which contains the PBMC, was collected with a Pasteur pipette into a new Falcon tube. PBMC were then washed with 15 mL PBS and centrifuged at 1750 RPM for 10 min with brake on. The supernatant was discarded, and washing was repeated with 15 mL PBS to remove platelets. After the second washing, supernatant was discarded and 2 mL ACK buffer was added to resuspend the pellet and remove any contaminating red blood cells. After incubating at room temperature for no more than 5 minutes, 10 mL R10 media were added and centrifuged for 5 min at 1750 RPM. The pellet was resuspended with R10, ready for counting.

#### Influenza virus/RSV stimulation of PBMC (cord, maternal and non-pregnant women blood samples)

PBMC from cord, maternal and non-pregnant women blood samples were isolated. In total, 2 × 10^5^ cells PBMC was incubated with either influenza virus or RSV at MOI 3 on 96-well U-bottom plate. The samples were incubated for 24 hours at 37 °C (5% CO_2_) before harvesting 200 µL supernatant in Eppendorf tubes and stored at −80 °C for further analysis.

#### Luminex assay

An in-house Luminex kit was used^46^. The standards were purchased from R&D systems with corresponding antibody pairs. Individual Luminex bead sets (Luminex, Riverside, CA) were coupled to cytokine-specific capture antibodies according to the manufacturer’s recommendations. 19 analytes were measured in this assay (IL-1α; IL-1β, IL-6, IL-12, TNF, IFNβ, IFNy, IL-2, IL-4, CCL2, CCL4, CCL5, CCL8, CXCL8, CXCL10, GM-CSF, TGFβ). Magnetic beads conjugated with capture antibody were diluted in Luminex assay buffer (PBS supplemented with 1% Goat serum, 1% mouse serum, 0.05% Tween 20 and 20 mM Tris-HCL). 50 µL of bead mix was put in a 96-well flat-bottomed plate with 50 µL undiluted samples or pre-diluted standards. Plates were incubated for 1.5 h on a plate shaker. Plates were then washed with washing buffer on a magnetic platform three times before adding 50 µL pre-diluted detection antibody cocktail. Plates were incubated for 1 h on a plate shaker as per the previous incubation step. The washing step was repeated, followed by the addition of 50 µL streptavidin-PE and incubation for 30 min on a plate shaker. The washing step was repeated, and 100 µL washing buffer was added to each well. The plate was shaken for another 10 min to thoroughly disperse the beads before reading the plate on the Bio-Plex®100 Luminex machine (BIO-RAD).

### Statistics

All statistical analyses were performed in Graph Pad Prism V9 (GraphPad Software, San Diego, CA), R version 3.5.0. A statistically significant difference was defined as a *P*-value < 0.05 by one-way analysis of variance (ANOVA).

## Supplementary information

Supplementary Information

## Data Availability

The RNA-Seq data will be submitted to a repository.
