# Supplementary material for: Modulating cytokine microenvironment during T cell activation induces protective RSV-specific lung resident memory T cells in early life in mice
Source: Npj Viruses. 2024 Dec 31;2:71. doi: 10.1038/s44298-024-00073-x (PMC11688237; doi:10.1038/s44298-024-00073-x)
Supplement: Supplementary file 1 — Supplementary Information [file 44298_2024_73_MOESM1_ESM.pdf]

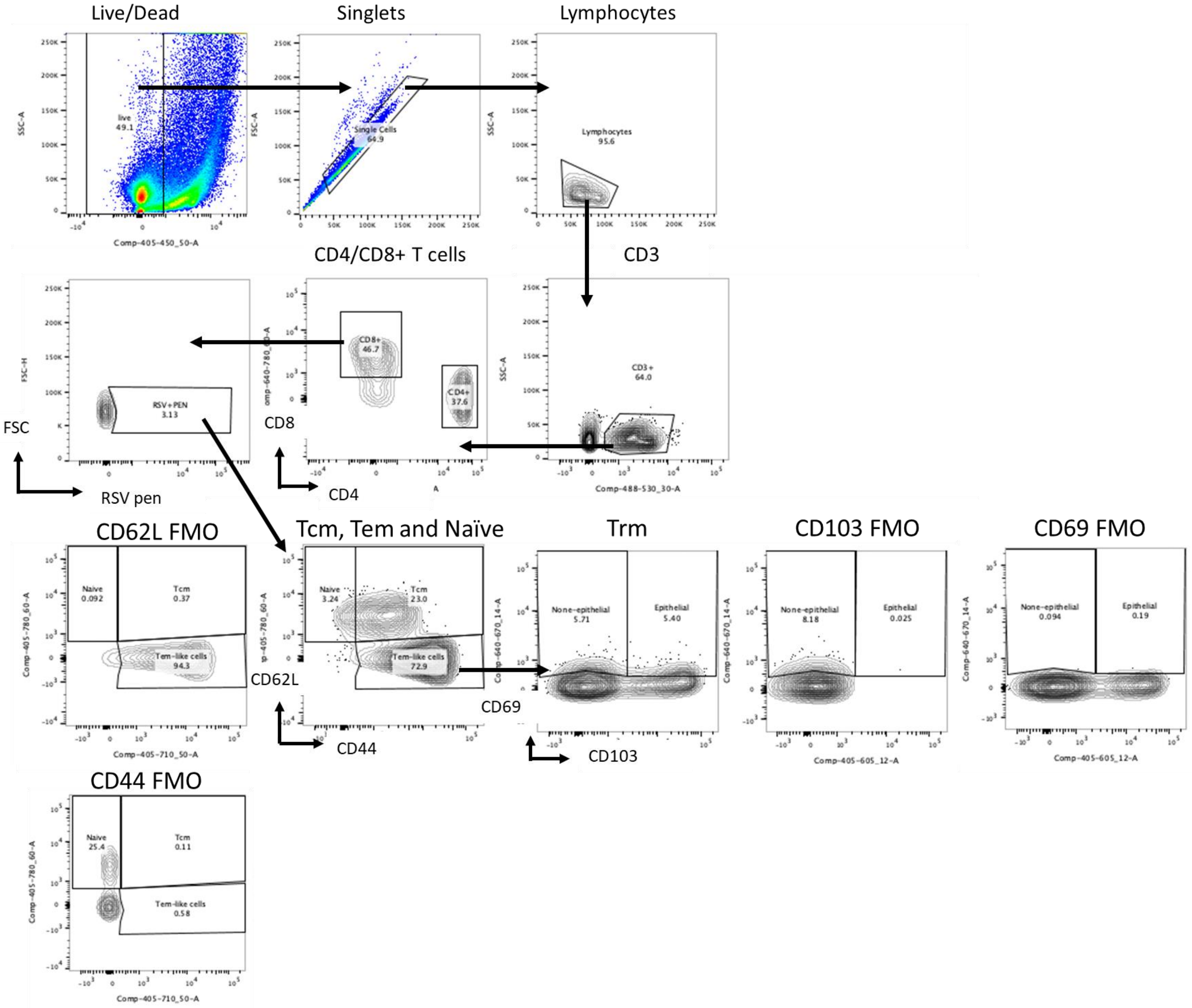

Fig S1. Gating strategy

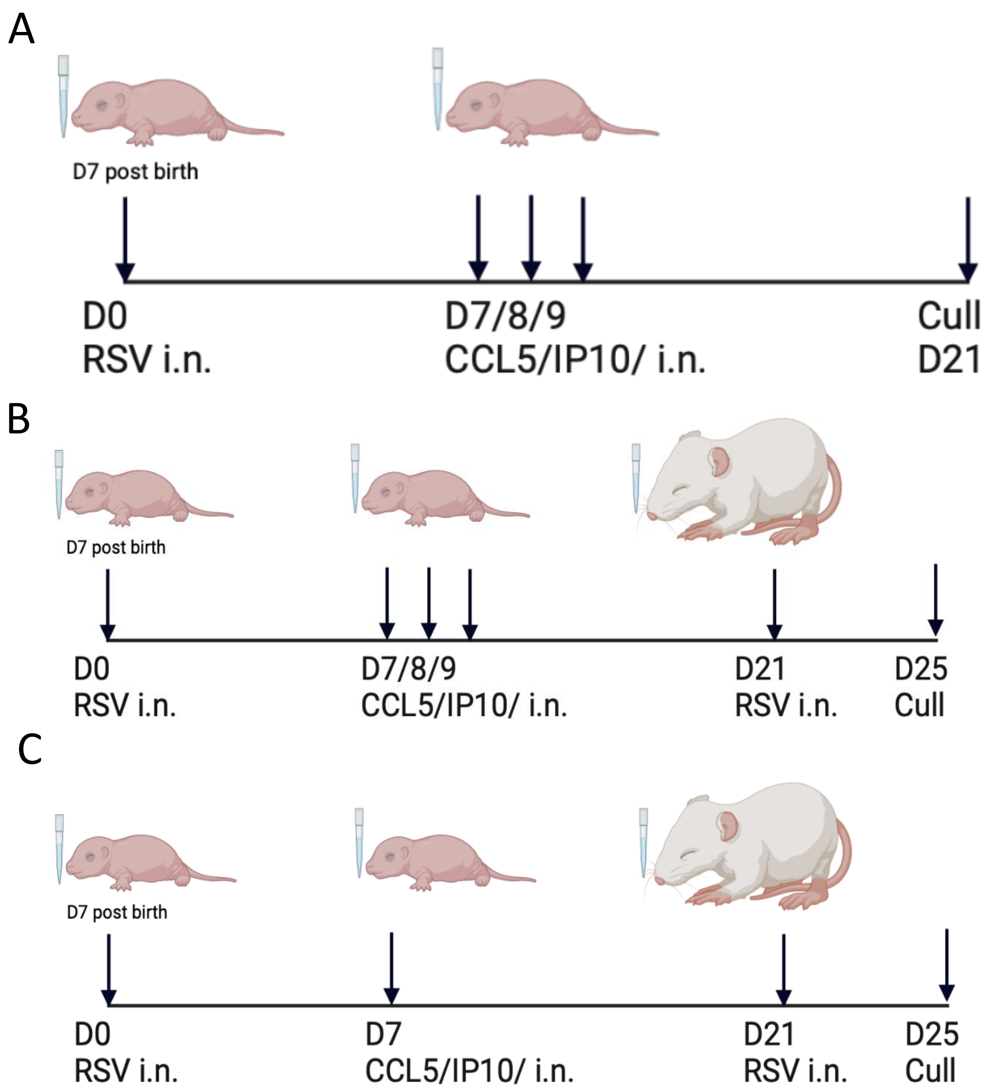

Fig S2. Chemokine boosting study in early life. Prime only study – infection with chemokine dosing d7-9 (A); Prime-challenge (B); Prime-Challenge, single chemokine dose (C).
